# Safety and feasibility of low‐dose ticagrelor in patients with ST‐segment elevation myocardial infarction

**DOI:** 10.1002/clc.23517

**Published:** 2020-12-15

**Authors:** Yanbo Wang, Yunfa Jiang, Wei Zhi, Yang Fu, Qing Wang, Jianli Zhou, Shaochen Zheng, Guozhen Hao

**Affiliations:** ^1^ Department of Cardiology Second Hospital of Hebei Medical University Shijiazhuang China

**Keywords:** low‐dose maintenance, major cardiac adverse events, platelet aggregation rate, ST‐segment elevation myocardial infarction, ticagrelor

## Abstract

**Background:**

Previous studies have confirmed the safety and feasibility of half‐dose ticagrelor in Chinese patients with acute coronary syndrome, but currently there is no plan for the use of ticagrelor for Chinese ST‐segment elevation myocardial infarction (STEMI) patients.

**Hypothesis:**

It is safe and feasible of low‐dose ticagrelor in patients with STEMI.

**Methods:**

The STEMI patients who were undergoing emergency intervention and taking ticagrelor were enrolled. Patients whose level of platelet aggregation rate (PAR) less than 30% after 7‐day treatment with standard‐dose ticagrelor were randomly divided into low‐dose group (LD group, 45 mg twice daily) and standard‐dose group (SD group, 90 mg twice daily). The changes of levels of platelet parameters were compared between the two groups. The incidence of major adverse cardiac events (MACE), bleeding events were compared between the two groups within 6 months of follow‐up.

**Results:**

The levels of PAR in the SD group decreased compared with baseline, and was lower than those of LD group at the same time point. The levels of platelet distribution width in both groups decreased from the baseline values (all *p* < .05) at 1, 3, and 6 months after grouping treatment, but there was no significant difference between the two groups. The incidence of MACE was similar between the two groups of patients. There were decreasing trends in the incidences of minimal bleeding event, minor bleeding event, dyspnea, and gout in the LD group.

**Conclusion:**

It is safe and feasible of low‐dose ticagrelor for patients with STEMI based on the monitoring of PAR.

ST‐segment elevation myocardial infarction (STEMI) is secondary thrombosis based on the rupture, erosion, erosion, and endothelial damage of unstable coronary plaque, leading to acute, continuous, and complete occlusion of coronary arteries. Excessive platelet activation and aggregation are central to the pathogenesis of STEMI. Ticagrelor is an oral, reversibly binding, direct‐acting P2Y_12_ receptor antagonist used clinically for the prevention of atherothrombotic events in patients with acute coronary syndromes (ACS).^1^ Compared with clopidogrel, the antiplatelet effect of ticagrelor is faster with stronger inhibition on platelet aggregation.^2^ The application of ticagrelor in STEMI patients has been unanimously recommended by many guidelines.^3^, ^4^, ^5^, ^6^ Importantly, East Asians show increased responses to ticagrelor compared with Caucasians.^7^ In recent years, individual treatment regimens of ticagrelor have received attention in Chinese patients. Previous studies have confirmed the safety and feasibility of half‐dose ticagrelor in Chinese patients with NST‐ACS, but currently there is no plan for the use of ticagrelor for Chinese STEMI patients. The purpose of this study was to explore the safety and feasibility of low‐dose ticagrelor in the treatment of STEMI based on the monitoring of platelet aggregation rate (PAR).

## 1 MATERIALS AND METHODS

This study is a single‐center, prospective, randomized controlled study. The STEMI patients who underwent emergency intervention in our chest pain center from January 2019 to December 2019 were enrolled. The research plan was approved by the Ethics Committee of the Second Hospital of Hebei Medical University, which was in line with the spirit of the Declaration of Helsinki. The informed consent was signed by all patients.

### 1.1 Inclusion criteria

Meeting the diagnosis for STEMI; within 12 h of onset; age 20–75 years; emergency intervention was performed; level of PAR <30% after 7 days of treatment with standard dose ticagrelor (180 mg loading dose, 90 mg twice daily); the informed consent was signed by all patients.

### 1.2 Exclusion criteria

Body weight <60 kg; a history of stroke or transient ischemic attack within 3 months; a history of gastrointestinal bleeding within 6 months; bleeding diathesis, platelet count <100 000/mm^3^, or hemoglobin <10 g/dl; hepatic dysfunction (serum liver enzyme or bilirubin >3 times the normal limit); renal insufficiency (serum creatine >2.5 mg/dl); severe chronic obstructive pulmonary disease; severe bradycardia (sick sinus syndrome or high‐degree atrioventricular block without pacemaker protection); drugs interfering with metabolism of ticagrelor.

### 1.3 Treatment strategy and grouping

After admission, patients were given oral loading doses of aspirin 300 mg and ticagrelor 180 mg. Following the guidelines and clinical path, the emergency coronary angiography was performed. According to the lesion of infarction related artery (IRA), the appropriate interventional treatment was performed by interventional cardiologist. The oral administration of aspirin 100 mg once daily and ticagrelor 90 mg twice daily was administered to all patients. Other therapeutic drugs such as heparin, low‐molecular‐weight heparin, β‐blockers, angiotensin converting enzyme inhibitor (ACEI) drugs, and statins are given in accordance with current guidelines. The level of PAR was rechecked 7 days after intervention, and patients who met the inclusion criteria were randomly divided into a low‐dose group (LD group, ticagrelor doses of 45 mg twice daily) and a standard‐dose group (SD group, ticagrelor doses of 90 mg twice daily).

PAR examination: The blood samples were centrifuged for 5 min at 120 g to obtain platelet‐rich plasma and further centrifuged for 10 min at 850 g to obtain platelet‐poor plasma. The platelet rich plasma and platelet‐poor plasma were stored at room temperature to be used within 2–3 h. The PAR was assessed by traditional light transmission aggregometry, which was performed as previously described.^8^ Briefly, platelets were stimulated with 5 μmol/L adenosine diphosphate (ADP). The PAR was expressed as the maximum percentage change in light transmittance from baseline, with platelet‐poor plasma as a reference.

The levels of platelet count (PLT), mean platelet volume (MPV), platelet distribution width (PDW) were measured by SYSMEX XE2100 automatic haematic analyzer.

### 1.4 Observation parameters

The clinical data including demographic data, previous medical history, and laboratory examinations were recorded and compared between the two groups. The changes of PAR, PLT, MPV, and PDW were compared between the two groups at the time of grouping, 1 week, 2 weeks, 1 month, 3 months, and 6 months after grouping. The incidence of major adverse cardiac events (MACE), bleeding events, and adverse reactions were compared between the two groups within 6 months of follow‐up. Bleeding complications were defined according to PLATO trail.^9^

### 1.5 Statistical methods

Statistical analysis of all data was performed using SPSS 22.0 software (IBM SPSS Statistics V22.0). Measurement data were expressed as mean ± *SD*, and *t* test was used for comparison between groups. Count data were expressed by numerical value and percentage, using *χ* ^2^ test (when the counting data was less than 5, Fisher's exact probability method was used for testing). Platelet parameters and PAR measured at different time points were tested using repeated analysis of variance. *p* < .05 considered the difference to be statistically significant.

## 2 RESULTS

From January 2019 to December 2019, a total of 284 cases of STEMI patients were admitted in our department, and 165 cases received ticagrelor treatment. A total of 71 cases of STEMI patients who met the inclusion criteria were admitted to our chest pain center. Among them, 3 patients refused to participate in the study, and patients who signed the informed consent were randomly divided into LD and SD group with 34 cases in each group. During follow‐up, 3 patients in the LD group and 2 patients in the SD group were lost to follow‐up or failed to complete the review as planned. Finally, 31 cases in LD group and 32 cases in SD group were enrolled.

### 2.1 Comparison of baseline characteristics between the two groups

There was no significant difference in clinical baseline data (age, gender, body mass index, previous medical history, myocardial infarction site, cardiac function grading, and baseline laboratory test results) between the two groups (all *p* > .05) (Table 1).

**TABLE 1:** Comparison of baseline characteristics between the two groups

	LD group (*n* = 31)	SD group (*n* = 32)	*p* value
Age (year)	55.65 ± 15.19	55.44 ± 11.0	.951
Male (%)	26(83.9)	27(84.4)	.956
BMI (kg/m^2^)	25.97 ± 2.87	25.60 ± 3.15	.621
History			
Hypertension (%)	19(61.3)	18(56.3)	.682
Diabetes (%)	8(25.8)	9(28.1)	.836
Stroke (%)	2(6.5)	3(9.4)	1.000
Onset‐FMC (h)	4.61 ± 2.05	4.71 ± 2.29	.855
Anterior wall MI (%)	17(54.8)	19(59.4)	.716
Killip classification (%)			.907
I	18(58.1)	17(53.1)	
II	10(32.3)	12(37.5)	
III	3(9.7)	3(9.4)	
Medications			
Aspirin (%)	31(100.0)	31(96.9)	1.000
Statins (%)	29(93.5)	31(96.9)	.613
β‐blocker (%)	25(80.6)	25(78.1)	.805
ACEI/ARB	24(77.4)	25(78.1)	.946
Lab examinations			
SCr (μmol/L)	69.50 ± 9.48	73.24 ± 11.30	.161
UA (μmol/L))	410.57 ± 30.13	402.83 ± 26.22	.281
Hb (g/L)	123.23 ± 12.47	128.94 ± 15.19	.109
PLT (×10^9^/L)	214.19 ± 34.09	227.94 ± 49.62	.206
MPV (fl)	8.51 ± 0.66	8.31 ± 1.16	.405
PDW (fl)	15.24 ± 1.92	14.85 ± 1.52	.368
PAR at admission (%)	78.94 ± 8.38	74.60 ± 11.73	.098
PAR before grouping (%)	22.03 ± 5.19	24.05 ± 5.22	.129
CRUSADE score	24.94 ± 4.78	23.00 ± 6.07	.166
Peak level of cTnI (ng/ml)	85.85 ± 11.68	84.29 ± 10.16	.574
Peak level of CK‐MB (U/L)	266.78 ± 50.93	256.92 ± 43.30	.411
LVEF (%)	50.82 ± 10.38	50.26 ± 8.72	.817

Abbreviations: BMI, body mass index; CK‐MB, creatine kinase‐MB; cTnI, cardiac troponin I; FMC, first medical contact; Hb, hemoglobin; LVEF, left ventricular ejection fraction; MPV, mean platelet volume; PAR, platelet aggregation rate; PDW, platelet distribution width; PLT, platelet; SCr, serum creatinine; UA, uric acid.

### 2.2 Comparison of intervention data between the two group

Both groups of patients underwent emergency intervention after admission. There was no significant difference between the two groups in the door‐balloon time, IRA distribution, TIMI blood flow grading before and after intervention, and stent implantation (all *p* > .05) (Table 2).

**TABLE 2:** Comparison of intervention data between the two groups

	LD group (*n* = 31)	SD group (*n* = 32)	*p* value
Door‐balloon (min)	70.51 ± 9.44	75.29 ± 10.06	.057
IRA distribution (%)			.926
LAD	17(54.8)	19(59.4)	
LCX	5(16.1)	5(15.6)	
RCA	9(29.0)	8(25.0)	
TIMI flow before intervention (%)			.967
0	13(41.9)	13(40.6)	
1	8(25.8)	9(28.1)	
2	6(19.4)	7(21.9)	
3	4(12.9)	3(9.4)	
Stent (/patient)	1.37 ± 0.28	1.37 ± 0.34	.968
TIMI flow after intervention (%)			1.000
≤2	3(9.7)	4(12.5)	
3	28(90.3)	28(87.5)	

Abbreviations: IRA, infarction related artery; LAD, left anterior descending; LCX, left circumflex; RCA, right coronary artery.

### 2.3 Changes of platelet parameters before and after grouping

Table 3 shows the changes in levels of PAR, PLT, MPV, and PDW before groups and at 1 week, 2 weeks, 1 month, 3 months, and 6 months after grouping. After grouping, the levels of PAR in LD group were similar to baseline (all *p* > .05). The levels of PAR in the SD group decreased compared with baseline (all *p* < .05), and was lower than those of LD group measured at the same time (all *p* < .05). There was no significant change in levels of PLT and MPV before and after treatment in both groups, and there was no significant difference between the two groups. It was found that the levels of PDW in both groups decreased from the baseline values (all *p* < .05) at 1 month, 3 months, and 6 months after grouping, but there was no significant difference between the two groups.

**TABLE 3:** Changes of platelet parameters before and after grouping

	LD group (*n* = 31)	SD group (*n* = 32)	*p* value
PAR0	22.03 ± 5.19	24.05 ± 5.22	.129
PAR1	21.12 ± 5.84^a^ ^b^	20.16 ± 3.18^a^	.422
PAR2	23.76 ± 5.77^a^ ^b^	17.96 ± 4.38^a^	0
PAR3	23.79 ± 4.98^a^ ^b^	20.03 ± 3.75^a^	.001
PAR4	22.05 ± 5.88^a^ ^b^	17.11 ± 2.59^a^	0
PAR5	22.97 ± 4.86^a^ ^b^	20.24 ± 1.81^a^	.004
PLT0	214.19 ± 34.09	227.94 ± 49.62	.206
PLT1	203.39 ± 43.69	219.88 ± 48.31	.155
PLT2	216.42 ± 38.67	238.56 ± 51.27	.058
PLT3	208.39 ± 34.30	216.25 ± 41.08	.414
PLT4	221.81 ± 48.00	223.69 ± 32.10	.855
PLT5	233.87 ± 50.21	237.41 ± 52.64	.786
MPV0	8.51 ± 0.66	8.31 ± 1.16	.405
MPV1	8.61 ± 0.76	8.56 ± 0.98	.836
MPV2	8.22 ± 0.95	8.57 ± 0.81	.120
MPV3	8.65 ± 1.03	8.70 ± 0.72	.799
MPV4	8.42 ± 0.72	8.69 ± 0.94	.207
MPV5	8.23 ± 0.69	8.46 ± 0.63	.170
PDW0	15.24 ± 1.92	14.85 ± 1.52	.368
PDW1	15.35 ± 2.59	14.81 ± 2.46	.403
PDW2	14.69 ± 1.69	14.32 ± 2.09	.440
PDW3	13.86 ± 1.78^a^	13.70 ± 2.05^a^	.740
PDW4	13.90 ± 1.77^a^	14.10 ± 1.65^a^	.645
PDW5	14.06 ± 1.73^a^	13.56 ± 1.83^a^	.273

*Note:* Time point 0: baseline; time point 1–5: 1 week, 2 weeks, 1 month, 3 months, and 6 months after grouping.

Abbreviations: MPV, mean platelet volume; PAR, platelet aggregation rate; PDW, platelet distribution width; PLT, platelet count.

^a^ Compared with the baseline level, *p* < .05.

^b^ Compared with the level of PAR in SD group at the same time point, *p* < .05.

### 2.4 Results of 6‐month follow‐up

The incidence of MACE was similar between the two groups of patients for 6 months of follow‐up (all *p* > .05). There were decreasing trends in the incidences of minimal bleeding event, minor bleeding event, dyspnea, and gout in the LD group (all *p* > .05) (Table 4).

**TABLE 4:** Results of 6‐month follow‐up

	LD group (*n* = 31)	SD group (*n* = 32)	*p* value
MACE(%)	3(9.7)	5(15.6)	.478
Recurrent angina (%)	1(3.2)	1(3.1)	1.000
Heart failure (%)	2(6.5)	3(9.4)	1.000
Death (%)	0(0)	1(3.1)	1.000
Bleeding complications (%)	4(12.9)	8(25.0)	.337
Minimal bleeding (%)	3(9.7)	6(18.8)	.474
Minor bleeding	1(3.2)	2(6.3)	1.000
Adverse responses (%)	2(6.5)	7(21.9)	.148
Dyspnea (%)	1(3.2)	4(12.5)	.355
Gout (%)	1(3.2)	3(9.4)	.613

## 3 DISCUSSION

STEMI is a common clinical cardiovascular emergency. In recent years, the incidence of STEMI has been increasing year by year. Platelet activation and aggregation is the initial link in the onset of STEMI, which has important significance in the onset of STEMI. Therefore, antiplatelet therapy is of great significance in STEMI treatment. Ticagrelor is the first reversibly binding, oral, direct‐acting P2Y_12_ receptor antagonist that binds reversibly and noncompetitively to the P2Y_12_ receptor. Recently, studies showed that standard‐dose ticagrelor has a more rapid onset of effect and a greater inhibition of platelet aggregation compared with high‐dose clopidogrel therapy.^10^, ^11^ At present, the application of ticagrelor in STEMI treatment has been unanimously recommended by many guidelines. However, standard‐dose ticagrelor is associated with a significant increase in the risk of bleeding, incidence of dyspnea^12^, ^13^, ^14^ and higher discontinuation rates due to adverse effects compared with clopidogrel. Studies have reported that a switch to a high‐dose of clopidogrel, ticagrelor, or prasugrel may be indicated.^15^ Previous studies demonstrated that a half‐dose of ticagrelor had an inhibitory effect on platelet aggregation that was better than clopidogrel^16^, ^17^, ^18^ and even that half‐dose ticagrelor produced a similar effect of platelet reactivity inhibition as standard‐dose ticagrelor in Chinese patients.^19^

However, there are still no relevant studies on low‐dose ticagrelor for STEMI patients. The results of this study found that it is safe and feasible to use low‐dose ticagrelor based on the monitoring of PAR.

The PAR induced by ADP is a commonly used to detect the effect of P2Y12 receptor inhibitors on platelet aggregation function. The detection is convenient and repeatable. In previously published studies, with 20 mM ADP as the agonist, mean inhibition of platelet aggregation observed with clopidogrel 300 mg PAR ranges from 20% to 40%.^20^, ^21^

The STEMI patients enrolled in this study had a good response on ticagrelor treatment and the levels of PAR were all of less than 30% after 7 days of treatment with standard dose ticagrelor, which ensured the effectiveness of the antiplatelet treatment. In this study, the level of PAR was also used as an important indicator for evaluating of effects of low‐dose ticagrelor treatment. In our study, although the levels of PAR in LD group were higher than the those in the SD group after grouping, it could still make platelet aggregation function maintain a low level without increasing the ischemic events during follow‐up. Meanwhile, there as a decreasing trend in incidences of bleeding events and other side effects in LD group. This result provides a reference for the individualized application of ticagrelor in Chinese STEMI patients. Due to the small sample size, further research is needed for observation and analysis.

PLT, MPV, and PDW are commonly used parameters for routine blood tests, which could reflect the platelet activation status and inflammatory response in STEMI patients.^22^, ^23^ In this study, after treatments with different doses of ticagrelor, the levels of PLT and MPV did not change significantly in both groups. This result may be related to the small sample size of the study, or it may be related to the limited sensitivity and specificity of MPV.^24^, ^25^ PDW, which is a novel marker for coronary artery disease, measures the variability of platelet size.^26^ Previous study has indicated that PDW and plateletcrit levels seem to be independent markers of STEMI in young patients and may reflect prothrombotic state in this specific population,^27^ and PDW could also be used as an early risk stratification of patients with acute myocardial infarction.^28^ In this study, after 1 month of treatment with different doses of ticagrelor, the PDW level of both groups of STEMI patients decreased significantly, suggesting that antiplatelet therapy could help reduce the platelet activation status and reduce the risk of ischemia in STEMI patients.

In conclusion, the results of this study found that the use of low‐dose ticagrelor is safe and feasible for patients with STEMI based on the monitoring of PAR. However, due to the small sample size and the relatively simple means of monitoring PAR, it is still necessary to expand the sample size and adopt a method such as thromboelastography to evaluate the strategy more comprehensively.

## CONFLICT OF INTEREST

The authors declare no potential conflict of interest.

## FUNDING INFORMATION

2019 Medical Research Projects of Hebei Province in China (20190523).

## Data Availability

No additional unpublished data are available.
